# Accuracy of Robot-Assisted Pedicle Screw Placement: Two-Center Experience with Learning Curve Analysis

**DOI:** 10.3390/jcm15145727

**Published:** 2026-07-22

**Authors:** Ismail Zaed, Carlo Brembilla, Giuseppe De Gennaro Aquino, Ernesto Pizzica, Jad El Choueiri, Leonardo Di Cosmo, Francesco Marchi, Ivan Cabrilo, Davide Milani, Andrea Cardia, Gabriele Capo

**Affiliations:** 1 Department of Neurosurgery, Neurocenter of South Switzerland, Ente Ospedaliero Cantonale, 6900 Lugano, Switzerland; francesco.marchi@eoc.ch (F.M.); ivan.cabrilo@eoc.ch (I.C.); davide.milani@eoc.ch (D.M.);; 2 Department of NESMOS, Sapienza University, 00185 Rome, Italy; 3 Department of Neurosurgery, IRCCS Humanitas Research Hospital, Via Alessandro Manzoni 56, 20089 Rozzano, Italy; carlo.brembilla@humanitas.it (C.B.); ernesto.pizzica@humanitas.it (E.P.); gabriele.capo@humanitas.it (G.C.); 4 Department of Biomedical Sciences, Humanitas University, Via Rita Levi Montalcini 4, 20090 Pieve Emanuele, Italyleonardo.dicosmo@st.hunimed.eu (L.D.C.)

**Keywords:** robotic, spine surgery, accuracy, pedicle screws

## Abstract

**Background**: Accurate pedicle screw placement remains essential in spinal instrumentation, and robotic navigation has been introduced to improve safety, reproducibility, and workflow standardization. This study evaluated the accuracy of robot-assisted pedicle screw placement using the Excelsius GPS platform during the first year of implementation at two centers and analyzed the associated learning curve. **Methods**: Consecutive patients undergoing robot-assisted spinal instrumentation between April 2024 and April 2025 were retrospectively reviewed. Screw accuracy was assessed on intraoperative three-dimensional imaging using the Gertzbein–Robbins Scale (GRS). Grades A and B were considered clinically acceptable, whereas grades C–E were considered clinically non-acceptable. Sacral S1 screws and oncological cases requiring carbon fiber-reinforced PEEK instrumentation were excluded from the primary analysis and evaluated separately when appropriate. Robotic workflow time was defined as the interval between the first intraoperative three-dimensional acquisition used for planning and the second acquisition used for screw verification. **Results**: The primary standard non-oncological cohort included 102 patients and 455 non-S1 screws. Overall, 411 screws were classified as GRS A, yielding a perfect intrapedicular placement rate of 90.3%. Clinically acceptable accuracy was achieved in 449 of 455 screws, corresponding to a GRS A + B rate of 98.7% (95% CI, 97.2–99.4%). Only six screws were classified as GRS C–E, with no GRS D screws observed. Clinically acceptable accuracy was comparable between centers. In Center 1, all clinically non-acceptable screws occurred within the first nine cases, and GRS A + B accuracy increased from 92.9% in the first trimester to 100% thereafter. Median robotic workflow time was 64.4 min per case and 13.9 min per screw. **Conclusions**: This two-center early experience supports the accuracy and reproducibility of ExcelsiusGPS-assisted spinal instrumentation. Chronological analysis showed that clinically non-acceptable breaches were concentrated in the early implementation phase; however, this observation should be considered exploratory because of the low event count. The study supports high clinically acceptable accuracy and broadly comparable robotic workflow metrics across centers. Chronological patterns observed during early implementation should be interpreted as exploratory rather than as proof of a formal learning curve.

## 1. Introduction

Pedicle screw fixation is a cornerstone of modern spinal surgery, but accurate screw placement remains technically demanding because of the variability in pedicle morphology, spinal deformity, degenerative changes, osteoporosis, and limited intraoperative visualization [1]. Malpositioned screws may lead to neurological, vascular, or visceral complications and may require revision surgery [1,2]. For these reasons, several image-guidance technologies have been introduced to improve the safety and reproducibility of spinal instrumentation [2].

Over the past two decades, spinal navigation and robotic-assisted platforms have progressively expanded the surgeon’s ability to plan and execute screw trajectories with high spatial accuracy [3]. The new generation of robotic navigation platform combines intraoperative image guidance with a rigid robotic arm that aligns the working channel according to the preplanned trajectory. The system allows screw trajectories to be planned on three-dimensional imaging and provides real-time navigated feedback during instrumentation. Importantly, the robotic platform does not autonomously place the screw; rather, it assists the surgeon by maintaining the planned trajectory while the surgeon performs drilling, tapping, and screw insertion [4].

Although robotic-assisted spinal instrumentation has been associated with high rates of clinically acceptable screw placement, the early implementation phase of a new robotic platform remains an important period to investigate [4]. In particular, it is unclear whether accuracy and operative workflow improve over time during the first institutional experience with the system [5]. Previous studies have reported high accuracy with the ExcelsiusGPS platform, but further single-center early-experience studies remain useful to better define the learning curve, reproducibility, and workflow implications of robotic-assisted screw placement [6,7].

The aim of this study was to evaluate the accuracy of robot-assisted pedicle screw placement using the ExcelsiusGPS platform during the first year of use at two different institutions. Screw accuracy was assessed using the Gertzbein–Robbins Scale, and the learning curve was evaluated by analyzing changes in clinically acceptable accuracy and robotic workflow time over consecutive time periods.

## 2. Material and Methods

### 2.1. Study Design and Patient Population

This was a retrospective study including consecutive patients who underwent robot-assisted spinal instrumentation with the ExcelsiusGPS robotic navigation platform at two different institutions, namely Neurocenter of South Switzerland and Humanitas Research Hospital between April 2024 and April 2025.

All patients who underwent robot-assisted screw placement during the study period were eligible for inclusion. Demographic data, diagnosis, surgical indication, type of procedure, number of implanted screws, screw level, screw side, Gertzbein–Robbins grade, and intraoperative timing data were collected from a prospectively maintained institutional database and retrospectively analyzed.

Patients were not excluded based on diagnosis or surgical approach, provided that robot-assisted instrumentation was performed. However, for the primary screw accuracy analysis, S1 screws were excluded, because S1 does not have a true pedicle in the same anatomical sense as thoracic and lumbar vertebrae. Screws inserted at levels treated with cement augmentation, kyphoplasty, or kyphoplasty-like procedures were not assigned a Gertzbein–Robbins grade when pedicle-based grading was not applicable. Although S1 screws were excluded from the primary pedicle-based analysis because of the distinct sacral anatomy and the limited applicability of pedicle-based GRS assessment at this level, they were analyzed separately and reported descriptively.

This study was conducted according to the “Ethical Principles for Medical Research Involving Human Subjects” stated in the Declaration of Helsinki issued in 2004, and its further revisions made in 2008 and 2013. To report our results, we followed the recommendations of the STROCCS (Strengthening the reporting of observational cohort studies in surgery) statement. Because of the retrospective design, the requirement for individual informed consent was waived according to institutional policy.

### 2.2. Robotic Platform and Surgical Workflow

All procedures were performed using the ExcelsiusGPS robotic navigation platform. The platform integrates three-dimensional imaging, surgical navigation, and a robotic arm designed to align the working channel with the planned screw trajectory. Preoperative or intraoperative imaging can be used for trajectory planning. In the present cohort, screw trajectories were planned after intraoperative three-dimensional acquisition. The robotic arm was then positioned according to the selected trajectory, and the surgeon performed the instrumentation through the robotic-guided working channel.

The standard workflow consisted of:

1. Patient positioning and exposure;
2. Placement of the dynamic reference frame;
3. Intraoperative three-dimensional imaging acquisition for navigation and planning;
4. Screw trajectory planning on the robotic workstation;
5. Robotic arm alignment to the planned trajectory;
6. Drilling, tapping, and screw insertion under robotic assistance;
7. Second intraoperative three-dimensional imaging acquisition for screw position verification.

The robot was used as a guidance and trajectory-alignment system; all drilling, tapping, and screw insertion steps were performed by the surgical team.

### 2.3. Timing Assessment

Robotic workflow time was defined as the interval between the first intraoperative three-dimensional acquisition used for screw planning and the second intraoperative three-dimensional acquisition used for screw position control. This interval was selected to capture the intraoperative robotic-navigation workflow related to screw planning, trajectory execution, and radiological verification. It was not intended to represent pure screw insertion time, total operative time, or skin-to-skin time. For each case, the following timing variables were calculated:

- Total robotic workflow time, in minutes;
- Robotic workflow time per screw, calculated as total robotic workflow time divided by the number of implanted screws.

When procedures such as kyphoplasty or kyphoplasty-like procedures were performed, these were completed before the first acquisition used for robotic planning; therefore, they were not included in the robotic workflow time interval.

### 2.4. Screw Accuracy Assessment

Screw accuracy was assessed on intraoperative control three-dimensional imaging using the Gertzbein–Robbins Scale (GRS). At each center, screw grading was performed separately by two study authors with experience in spinal instrumentation assessment. In cases of uncertainty or disagreement, the corresponding senior author of the respective center reviewed the imaging and adjudicated the final classification.

The grading process was performed locally at each institution and was not centrally standardized across centers. Reviewers were not formally blinded to center or chronological case order, and formal interobserver reliability statistics were not calculated.

The grading system was defined as follows: grade A, screw completely contained within the pedicle; grade B, cortical breach < 2 mm; grade C, cortical breach ≥ 2 mm and <4 mm; grade D, cortical breach ≥ 4 mm and <6 mm; and grade E, cortical breach ≥ 6 mm.

For the primary analysis, grades A and B were considered clinically acceptable, whereas grades C–E were considered clinically non-acceptable. The primary accuracy endpoint was the proportion of non-S1 screws classified as GRS A + B. Secondary endpoints included the proportion of GRS A screws and the distribution of individual GRS grades.

### 2.5. Oncological Cases

Oncological cases at our institution were treated with carbon fiber-reinforced PEEK instrumentation with the use of ExcelsiusGPS robotic navigation platform, in a technique previously discussed [8,9]. Since the use of carbon fiber-reinforced PEEK screws with the robotic system is to be considered “off-label”, they were analyzed separately. In spinal oncology, CFR-PEEK screws are used to minimize imaging artifacts and facilitate postoperative radiotherapy planning and tumor surveillance, and their radiological assessment may differ from standard titanium screws. Moreover, tumor-related bone destruction, previous or planned radiotherapy, and complex reconstructive requirements may influence both screw trajectory planning and accuracy.

The oncological subgroup included cases from both centers, comprising one patient with four non-S1 screws from Center 1 and seven patients with 42 non-S1 screws from Center 2.

### 2.6. Chronological Implementation Analysis

Temporal changes in accuracy and robotic workflow time were evaluated as exploratory institutional implementation analyses rather than as formal proficiency curve analyses. The chronological distribution of clinically non-acceptable screws, defined as GRS C–E, was examined descriptively. Because of the low number of clinically non-acceptable events, no multivariable models were fitted for GRS C–E placement.

Accuracy-related temporal findings were interpreted with attention and were not used to define a formal learning threshold. The potential influence of unmeasured factors, including case complexity, spinal deformity, revision status, anatomical variability, and case selection, was acknowledged. For workflow analysis, robotic workflow time and robotic workflow time per screw were evaluated across chronological case sequence and consecutive time periods.

### 2.7. Statistical Analysis

Continuous variables were summarized as mean ± standard deviation or median with interquartile range, depending on distribution. Categorical variables were summarized as counts and percentages. The proportion of screws classified as GRS A and GRS A + B was calculated for the overall cohort and for each learning curve period. Exact or Wilson 95% confidence intervals were calculated for key proportions.

Comparisons of categorical variables, including GRS A versus non-A and GRS A + B versus GRS C–E, were performed using Fisher’s exact test because of the small number of non-acceptable breaches.

Continuous variables, including robotic workflow time and robotic workflow time per screw, were compared between groups using the Mann–Whitney U test or Kruskal–Wallis test, where appropriate. Correlations between chronological case order and timing variables were assessed using Spearman’s rank correlation coefficient. Analyses were performed with R version 4.1.1 (R Foundation for Statistical Computing, Vienna, Austria). A two-sided *p* value < 0.05 was considered statistically significant.

## 3. Results

### 3.1. Study Population

A total of 102 standard non-oncological patients from two centers were included in the primary multicenter analysis of screw accuracy. Center 1 contributed 74 patients, while Center 2 contributed 28 patients. Overall, 455 non-sacral screws were included in the main Gertzbein–Robbins Scale analysis. Oncological cases requiring carbon fiber-reinforced PEEK instrumentation were excluded from the primary analysis and were evaluated separately as an exploratory subgroup.

### 3.2. Screw Accuracy

In the combined standard non-oncological cohort, 411 of 455 screws were classified as GRS A, corresponding to a perfect intrapedicular placement rate of 90.3%. An additional 38 screws were classified as GRS B. Therefore, 449 of 455 screws were classified as GRS A + B, resulting in a clinically acceptable accuracy rate of 98.7% (95% CI, 97.2–99.4%). All data have been summarized in Table 1.

Only six screws were classified as GRS C–E, corresponding to a clinically non-acceptable breach rate of 1.3%. No GRS D screws were observed in the combined standard cohort.

Clinically acceptable accuracy was similar between centers. Center 1 achieved 98.8% GRS A + B accuracy, while Center 2 achieved 98.4% GRS A + B accuracy. There was no statistically significant difference between centers for clinically acceptable screw placement.

A total of 46 S1 screws were excluded from the primary non-S1 analysis. Thirty-six were placed in Center 1 and ten in Center 2. Overall, 45 screws were classified as GRS A and one as GRS B; no GRS C–E S1 screws were observed. Clinically acceptable S1 screw placement was achieved in all excluded S1 screws (46/46, 100%). These screws will not be included in the final analysis.

**Figure 1:**
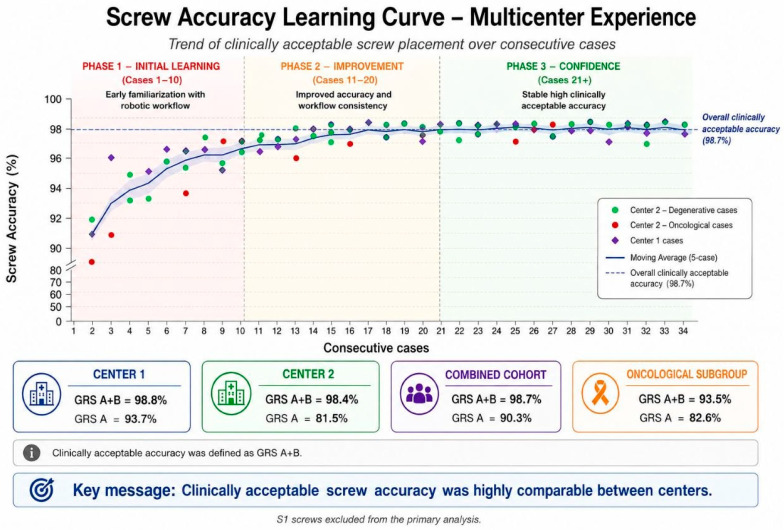
Learning curve of screw accuracy.

By contrast, the rate of perfect intrapedicular screws differed between centers. GRS A accuracy was 93.7% in Center 1 and 81.5% in Center 2. However, when looking at the clinically acceptable results (A + B), there were no statistical differences among the two centers. The results have been summarized in Figure 1.

### 3.3. Robotic Workflow Event

Robot abandonment was defined as discontinuation of robot-assisted instrumentation after workflow initiation, requiring completion of instrumentation with an alternative technique. No robot abandonment was recorded during the study period.

No intraoperative adverse events attributable to robotic system malfunction were documented. Screw position was assessed by intraoperative three-dimensional verification imaging; malpositioned screws were recorded according to the Gertzbein–Robbins classification.

### 3.4. Learning Curve Analysis

The learning curve was assessed using accuracy-based and time-based endpoints. For the present analysis, the accuracy-based learning curve was evaluated using the chronological case sequence and the observed distribution of GRS C–E screws. All data have been summarized in Table 1 and Table 2.

In the combined standard cohort, six clinically non-acceptable screws were observed. Of these occurring in Centers 1 and 2, four occurred in Center 2. The low number of GRS C–E screws limited the possibility of performing robust multivariable modeling, but the overall breach rate remained low across both centers.

In Center 1, all clinically non-acceptable screws occurred within the first nine cases. This chronological clustering was described as an exploratory observation and was not interpreted as evidence of a definitive proficiency threshold, given the low number of GRS C–E events. No adjusted analysis of GRS C–E placement was performed because only six clinically non-acceptable screws occurred in the primary cohort, precluding reliable multivariable modeling.

### 3.5. Timing Analysis

In Center 1, robotic workflow time was available and was defined as the interval between the first intraoperative three-dimensional acquisition used for planning and the second intraoperative three-dimensional acquisition used for screw position control. All data have been summarized in Table 3.

In Center 1, the median robotic workflow time was 63.0 min per case (interquartile range [IQR], 49.5–88.5 min) and 13.8 min per screw (IQR, 10.9–17.3 min/screw). Robotic workflow time showed no statistically significant monotonic association with chronological case order, either when expressed per case (Spearman’s rho = −0.181, *p* = 0.120) or per screw (Spearman’s rho = −0.160, *p* = 0.169). For Center 2, the median robotic workflow time was 65.3 min per case (IQR, [53.2–97.1] min) and 14.2 min per screw (IQR, [11.3–18.8] min/screw). Chronological case order showed [no/a statistically significant] monotonic association with robotic workflow time per case.

### 3.6. Oncological Subgroup

The exploratory oncological subgroup included eight patients and 46 non-S1 screws, comprising one patient from Center 1 and seven patients from Center 2. Oncological cases requiring carbon fiber-reinforced PEEK instrumentation were analyzed separately because they represented a distinct technical and clinical subgroup. This exploratory subgroup included eight patients and 46 non-S1 screws. All data have been summarized in Table 4 and Table 5.

In this subgroup, 38 screws were classified as GRS A and five screws as GRS B. Therefore, 43 of 46 screws were classified as GRS A + B, corresponding to 93.5% clinically acceptable accuracy. Three screws were classified as GRS C, and no GRS D or E screws were observed.

Given the small sample size and the distinct characteristics of oncological instrumentation, this subgroup was not pooled with the standard cohort for the primary analysis.

## 4. Discussion

This two-center early experience demonstrates that robotic-assisted spinal instrumentation can achieve high clinically acceptable screw accuracy during the initial phase of institutional adoption. In the combined standard non-oncological cohort, 449 of 455 non-sacral screws were classified as GRS A + B, corresponding to a clinically acceptable accuracy rate of 98.7%. Only 1.3% of screws were classified as GRS C–E, and no GRS D screws were observed.

The most relevant finding of this study is the consistency of clinically acceptable accuracy across two independent centers. Center 1 achieved 98.8% GRS A + B accuracy, while Center 2 achieved 98.4% GRS A + B accuracy. This near-identical rate of clinically acceptable screw placement suggests that robotic-assisted fixation may provide a reproducible safety profile across different surgical teams and institutional workflows.

The distinction between GRS A and GRS A + B is important when interpreting these findings. Center 1 had a higher rate of perfect intrapedicular screws than Center 2, with GRS A rates of 93.7% and 81.5%, respectively. However, the clinically acceptable accuracy rate was comparable between centers because Center 2 had a higher proportion of GRS B screws. This suggests that minor cortical breaches below 2 mm may vary between centers because of differences in anatomy, grading interpretation, imaging quality, screw trajectory planning, or institutional workflow, without necessarily translating into a clinically meaningful difference in safety [10,11]. The difference in GRS A rates between centers should be interpreted cautiously. Although Center 1 showed a higher proportion of perfect intrapedicular screws, the clinically acceptable GRS A + B rate was comparable between centers. This difference may reflect anatomical, imaging-related, workflow-related, or technical factors; however, it may also partly reflect measurement variability because grading was performed locally at each center, without centralized blinded adjudication or formal interobserver reliability testing.

From a clinical perspective, the avoidance of GRS C–E screws is arguably more relevant than the isolated proportion of GRS A screws. GRS B screws are generally considered clinically acceptable because the breach is less than 2 mm. Therefore, the present data suggest that the robotic platform may be particularly effective in minimizing clinically relevant breaches, even if minor deviations from a complete intrapedicular trajectory may still occur.

The learning curve analysis supports this interpretation. In Center 1, all clinically non-acceptable screws occurred during the early implementation phase. When the cohort was divided into consecutive three-month periods, clinically acceptable accuracy improved from 92.9% in the first trimester to 100% in each subsequent trimester. This finding suggests that the accuracy-based learning curve was not characterized by a slow linear increase in GRS A screws, but rather by the rapid disappearance of clinically relevant breaches after the initial phase of robotic adoption [12]. Nevertheless, the findings provide practical information for centers adopting robotic spinal instrumentation, suggesting that early cases may warrant particular attention to workflow standardization, image acquisition, registration, trajectory planning, and verification. Although robotic guidance may reduce technical variability through trajectory planning and rigid alignment, the present study cannot determine whether accuracy or workflow is independent of surgeon experience.

This pattern has important implications. In a robotic workflow, once the surgical team becomes familiar with patient registration, image acquisition, trajectory planning, robotic arm positioning, and navigated instrumentation, the system may help standardize screw placement and reduce major deviations. Thus, the learning curve may be better captured by the reduction in GRS C–E screws than by changes in the already high overall rate of clinically acceptable placement.

The timing analysis adds a further dimension to the learning curve: interval between the first planning acquisition and the second control acquisition. In the combined cohort, the median robotic workflow time was 64.4 min per case and 13.9 min per screw. Center-specific values were very similar, with 63.0 min per case and 13.8 min per screw in Center 1 and 65.3 min per case and 14.2 min per screw in Center 2.

This similarity is relevant because it suggests that the robotic workflow is not only accurate but also temporally reproducible across centers when measured using a standardized definition. Robotic workflow time is a more specific metric than total operative time because it focuses on the phase of surgery directly related to robotic planning, screw insertion, and intraoperative verification. Total operative time, by contrast, is influenced by exposure, decompression, interbody fusion, osteotomies, closure, anesthesiologic factors, and case complexity.

However, robotic workflow time should not be interpreted as pure screw insertion time. It includes image acquisition, trajectory planning, robotic alignment, screw placement, and verification imaging. For this reason, the term “robotic workflow time” is preferable to “screw placement time”. This distinction should be clearly stated because it makes the timing endpoint more methodologically robust and avoids overinterpretation.

The time-based findings should be interpreted with attention. Robotic workflow time captures a clinically relevant portion of the robotic pathway but does not isolate robotic execution alone. This interval includes image acquisition, registration, trajectory planning, robotic arm positioning, screw insertion, and verification imaging, and may also be influenced by the number of screws, surgical exposure, concurrent decompression or fusion procedures, team familiarity, imaging workflow, and case complexity.

No statistically significant chronological association was observed between case order and robotic workflow time, either per case or per screw. Therefore, the present data do not establish a time-based learning curve. Rather, they provide a descriptive estimate of robotic workflow duration during early institutional implementation.

Although median workflow times were numerically comparable between centers, this observation should not be interpreted as definitive evidence of workflow reproducibility. The study was not designed or powered to demonstrate equivalence between centers, and workflow duration may be affected by unmeasured differences in case mix, procedure type, number of instrumented levels, surgical exposure, and institutional organization.

From a practical perspective, the early clustering of clinically non-acceptable screws may be relevant for centers introducing robotic spinal instrumentation. Although the present study cannot define a formal proficiency threshold, it suggests that the earliest implementation phase may warrant enhanced supervision, standardized workflow protocols, and careful verification of image acquisition, registration, and trajectory planning. Prospective studies incorporating case-complexity adjustment and predefined competency metrics are needed to establish evidence-based training and credentialing thresholds.

The separate analysis of oncological/off-label cases is another important methodological point. Patients undergoing surgery for spinal tumors often present with altered anatomy, osteolytic bone destruction, previous or planned radiotherapy, and complex reconstructive requirements. Furthermore, carbon fiber-reinforced PEEK screws are used for different reasons than standard titanium screws, particularly to reduce imaging artifacts and facilitate postoperative tumor surveillance and radiotherapy planning. For these reasons, oncological CFR-PEEK screw cases were considered a distinct subgroup and were excluded from the primary standard cohort analysis.

In the exploratory oncological subgroup, clinically acceptable accuracy was 93.5%, with 43 of 46 screws classified as GRS A + B. Although this rate remained high, it was lower than the 98.7% observed in the standard cohort. This difference should be interpreted with caution because the oncological subgroup was small and likely included more complex anatomical conditions. Nevertheless, the finding supported the methodological decision to treat oncological instrumentation as a separate subgroup.

### Study Limitations

This study has several limitations. First, the retrospective design may introduce selection and information bias. Second, screw grading was based on radiological assessment, and formal interobserver reliability was not evaluated. Third, operator identity was not consistently recorded, preventing assessment of surgeon-specific learning curves. Finally, the low number of clinically non-acceptable screws limits the possibility of robust multivariable modeling.

Despite these limitations, the present multicenter analysis supports the accuracy and reproducibility of ExcelsiusGPS-assisted spinal instrumentation. The learning curve appeared to be driven mainly by the early disappearance of clinically relevant breaches.

The low number of clinically non-acceptable screws limited the statistical power for accuracy-based temporal analyses and precluded reliable multivariable modeling of GRS C–E placement. Furthermore, potentially relevant determinants of screw accuracy, including deformity severity, revision status, anatomical complexity, bone quality, and case-specific procedural difficulty, were not uniformly captured across both databases. Therefore, the chronological distribution of clinically non-acceptable screws should not be interpreted as proof of a formal learning curve or a fixed proficiency threshold.

The available dataset did not uniformly capture all potential determinants of workflow duration and screw accuracy, including deformity severity, revision status, surgical exposure, concurrent decompression or interbody fusion, bone quality, and detailed case complexity. Therefore, fully adjusted time-based learning curve modeling was not feasible.

The study did not include a contemporaneous freehand, navigation-only, or pre-robot institutional comparator. Consequently, the present findings describe early robotic implementation but do not establish superiority over other instrumentation techniques. Finally, patient-level clinical outcomes, radiation exposure, revision surgery, and complication rates were not systematically analyzed and should be investigated in future prospective comparative studies.

Surgeon identity, prior robotic and non-robotic spinal instrumentation experience, minimally invasive versus open approach, revision status, deformity diagnosis, body mass index, osteoporosis, screw dimensions, registration failures, and detailed procedure-specific complexity variables were not uniformly captured across the two databases. These factors may influence both screw accuracy and robotic workflow duration and could not be included in adjusted analyses.

## 5. Conclusions

In conclusion, this two-center early experience supports the accuracy and reproducibility of ExcelsiusGPS-assisted spinal instrumentation in standard non-oncological cases. In the combined cohort, 98.7% of screws were classified as GRS A + B, with comparable clinically acceptable accuracy between centers. The learning curve was mainly reflected by the early disappearance of clinically relevant breaches, while harmonized timing analysis showed similar robotic workflow duration across centers.

## Figures and Tables

**Table 1:** Screw accuracy in the standard non-oncological cohort.

Variable	Center 1	Center 2	Combined Cohort
Patients	74	28	102
Non-S1 screws	331	124	455
GRS A	310/331, 93.7%	101/124, 81.5%	411/455, 90.3%
GRS B	17/331, 5.1%	21/124, 16.9%	38/455, 8.4%
GRS C	3/331, 0.9%	2/124, 1.6%	5/455, 1.1%
GRS D	0/331, 0%	0/124, 0%	0/455, 0%
GRS E	1/331, 0.3%	0/124, 0%	1/455, 0.2%
GRS A + B	327/331, 98.8%	122/124, 98.4%	449/455, 98.7%
GRS C–E	4/331, 1.2%	2/124, 1.6%	6/455, 1.3%

**Table 2:** Accuracy in consecutive three-month period.

Period	Non-S1 Screws	GRS A	GRS A + B	GRS C–E
Trimester 1	56	49/56, 87.5%	52/56, 92.9%	4
Trimester 2	84	79/84, 94.0%	84/84, 100%	0
Trimester 3	76	74/76, 97.4%	76/76, 100%	0
Trimester 4	119	112/119, 94.1%	119/119, 100%	0

**Table 3:** Robotic workflow timing analysis.

Variable	Center 1	Center 2	Combined
Timing definition	First acquisition to second acquisition	First acquisition to second acquisition	First acquisition to second acquisition
Patients	74	28	102
Median robotic workflow time per case	63.0 min	65.3 min	64.4
Median robotic workflow time per screw	13.8 min/screw	14.2 min/screw	13.9 min/screw

**Table 4:** Exploratory oncological subgroup.

Variable	Oncological Subgroup (%)
Patients	8
Screws placed	46
GRS A	38/46, 82.6%
GRS B	5/46, 10.9%
GRS C	3/46, 6.5%
GRS D	0/46, 0%
GRS E	0/46, 0%
GRS A + B	43/46, 93.5%
GRS C–E	3/46, 6.5%

**Table 5:** Screw accuracy according to spinal region and oncological status.

Cohort	Spinal Region	Non-S1 Screws	GRS A	GRS B	GRS C	GRS D	GRS E	GRS A (%)	GRS A + B (%)	GRS C–E (%)
Standard non-oncological	Cervical	0	0	0	0	0	0	—	—	—
Standard non-oncological	Thoracic	103	99	4	0	0	0	96.1%	100%	0%
Standard non-oncological	Lumbar	352	312	34	5	0	1	88.6%	98.3%	1.7%
Standard non-oncological total	All regions	455	411	38	5	0	1	90.3%	98.7%	1.3%
Oncological subgroup	Cervical	10	7	0	3	0	0	70.0%	70.0%	30.0%
Oncological subgroup	Thoracic	24	24	0	0	0	0	100%	100%	0%
Oncological subgroup	Lumbar	12	7	5	0	0	0	58.3%	100%	0%
Oncological subgroup total	All regions	46	38	5	3	0	0			

## Data Availability

The original contributions presented in this study are included in the article. Further inquiries can be directed to the corresponding author.
